# Supplementary material for: Spinel rGO Wrapped CoV2O4 Nanocomposite as a Novel Anode Material for Sodium-Ion Batteries
Source: Polymers (Basel). 2020 Mar 3;12(3):555. doi: 10.3390/polym12030555 (PMC7182827; doi:10.3390/polym12030555)
Supplement: Supplementary file 1 [file polymers-12-00555-s001.pdf]

# Supporting information

# Spinel rGO wrapped $CoV_2O_4$ nanocomposite as a novel anode material for sodium-ion batteries

Rasu Muruganantham, Jeng-Shin Lu and Wei-Ren Liu*

Department of Chemical Engineering, R&D Center for Membrane Technology, Research Center for Circular Economy, Chung Yuan Christian University, 200 Chung Pei Road, Chung Li District, Taoyuan City, Taiwan 32023, ROC.
*Corresponding author. Tel: +886-3-2653315; Fax: 886-3-2653399
E-mail: WRLiu1203@gmail.com (W. R. Liu)

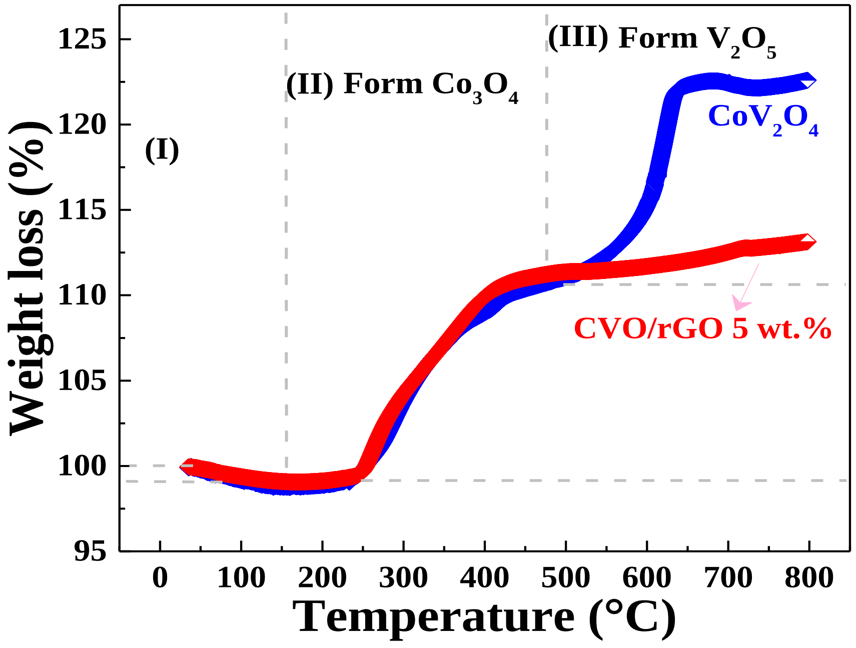


**Fig. S1** TGA analysis of synthesized bare $CoV_2O_4$ and rGO-wrapped $CoV_2O_4$ materials.

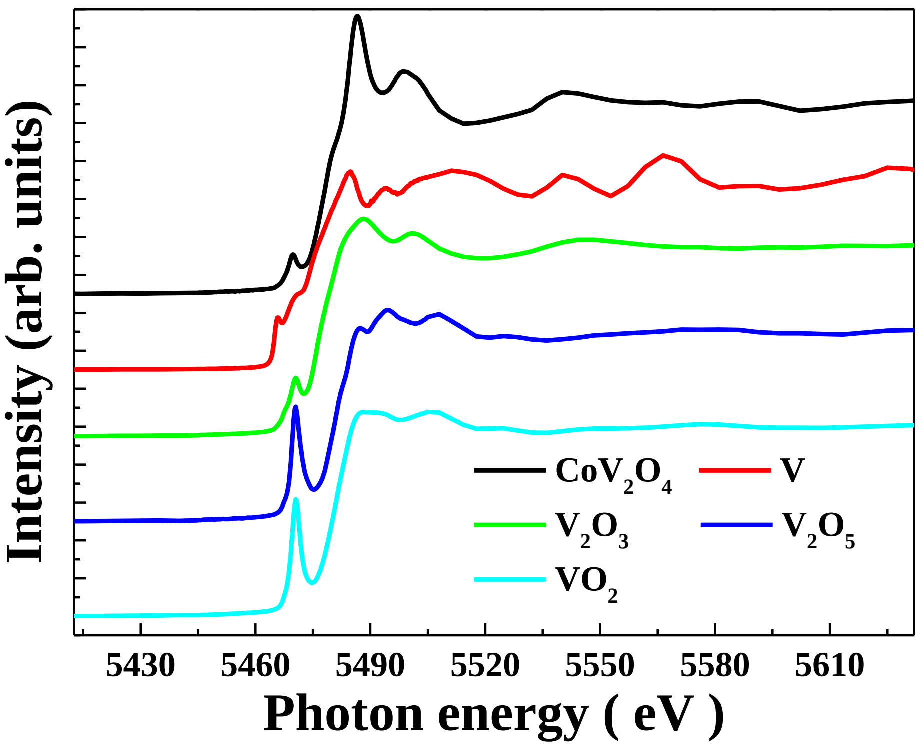


**Fig. S2** V *K*-edge X-ray absorption near edge structure spectroscopy (XANES) of $CoV_2O_4$ powder and the references of V, $VO_2$, $V_2O_3$, and $V_2O_5$ spectra.

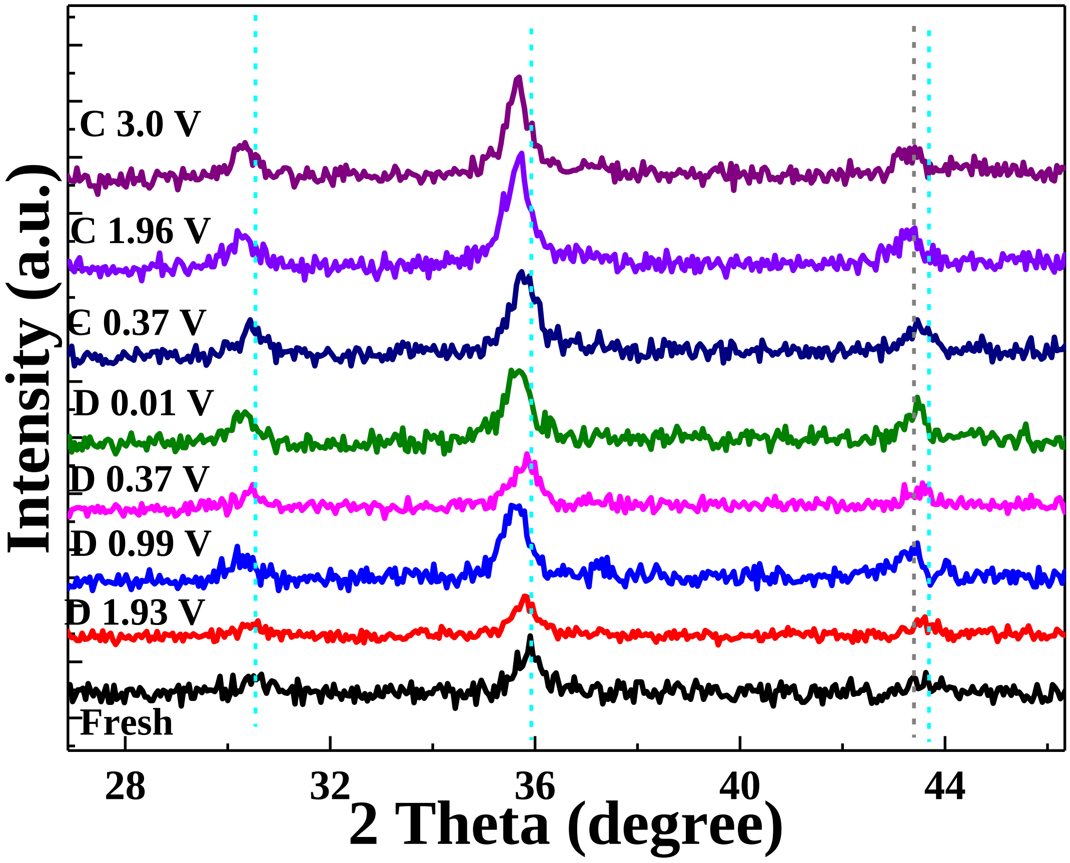


**Fig. S3** Enlarge 2θ values of *ex-situ* XRD analysis of CVO electrode cells.
